# Comparative co-expression analysis of RNA-Seq transcriptome revealing key genes, miRNA and transcription factor in distinct metabolic pathways in diabetic nerve, eye, and kidney disease

**DOI:** 10.5808/gi.22029

**Published:** 2022-09-30

**Authors:** Veerankutty Subaida Shafna Asmy, Jeyakumar Natarajan

**Affiliations:** Data Mining and Text Mining Laboratory, Department of Bioinformatics, Bharathiar University, Coimbatore, Tamilnadu 641 046, India

**Keywords:** co-expression analysis, diabetes nephropathy, diabetes neuropathy, diabetes retinopathy, microvascular complication, RNA-Seq transcriptome

## Abstract

Diabetes and its related complications are associated with long term damage and failure of various organ systems. The microvascular complications of diabetes considered in this study are diabetic retinopathy, diabetic neuropathy, and diabetic nephropathy. The aim is to identify the weighted co-expressed and differentially expressed genes (DEGs), major pathways, and their miRNA, transcription factors (TFs) and drugs interacting in all the three conditions. The primary goal is to identify vital DEGs in all the three conditions. The overlapped five genes (*AKT1*, *NFKB1*, *MAPK3*, *PDPK1*, and *TNF*) from the DEGs and the co-expressed genes were defined as key genes, which differentially expressed in all the three cases. Then the protein-protein interaction network and gene set linkage analysis (GSLA) of key genes was performed. GSLA, gene ontology, and pathway enrichment analysis of the key genes elucidates nine major pathways in diabetes. Subsequently, we constructed the miRNA-gene and transcription factor-gene regulatory network of the five gene of interest in the nine major pathways were studied. hsa-mir-34a-5p, a major miRNA that interacted with all the five genes. RELA, FOXO3, PDX1, and SREBF1 were the TFs interacting with the major five gene of interest. Finally, drug-gene interaction network elucidates five potential drugs to treat the genes of interest. This research reveals biomarker genes, miRNA, TFs, and therapeutic drugs in the key signaling pathways, which may help us, understand the processes of all three secondary microvascular problems and aid in disease detection and management.

## Introduction

Diabetes mellitus (DM) is a metabolic disorder characterized by hyperglycemia that results from a defect in insulin secretion, insulin action, or both [1]. Hyperglycaemia over the long term adversely impacts the microvasculature, leading to diabetic nephropathy (DN), diabetic retinopathy (DR), and diabetic neuropathy (DPN) with profound impact on the quality of life and life expectancy [2]. DM is on the rise globally. According to the statistics from International Diabetes Federation (accessed December 23, 2021) 90 million adults (20-79) are living with diabetes in the IDF South-East Asia (SEA) region in 2021 [3]. This figure is estimated to increase to 113 million by 2030 and 152 million by 2045.

As diabetes prevalence rises and the clinical arsenal for primary and secondary prevention of these complications expands. It is important for physicians to understand the relationship between diabetes and vascular disease. Nephropathy, retinopathy, and neuropathy are exacerbated by microvascular lesions, and cardiovascular events are associated with large vascular damage. This study examined the microvascular complications of diabetes, including DR, DN, and DPN. Both the duration and severity of hyperglycemia increases the risk of DR and other microvascular complications [4].

Increased glucose influx activates cellular signaling pathways such as the diacylglycerol-protein kinase C (PKC) pathway, advanced glycation end-products (AGE), polyol pathway, hexosamine pathway, and oxidative stress. Among diabetic microvascular complications, DR may be the most common. DR is characterized by four major mechanisms: increased polyol pathway flux, increased AGE formation, activation of PKC, and polyol pathways [5]. In case of other complications of diabetes, prevention is the first step in treating diabetic nephropathy. Diabetes microvascular complications, such as diabetic nephropathy, have strong associations with blood glucose control. An increase in glucose metabolic flux results in the activation of several metabolic pathways, resulting in an increase in AGE and reactive oxygen species production. This activates a number of signaling pathways that lead directly to enhanced extracellular matrix production via PKC-β stimulation of AP-1 transcriptional activation, ERK pathways, and, critically, transforming growth factor β1 (TGF-β1) synthesis, which then stimulates its signaling pathways to enhance extracellular matrix protein synthesis [6].

Despite the exact mechanism of hyperglycemia-induced nerve damage is unknown; it is believed to be caused by polyol accumulation, AGE damage, and oxidative stress. Although there is no specific treatment for diabetic neuropathy, there are many drugs that can alleviate its symptoms. Controlling symptoms and preventing worsening of neuropathy are the primary goals of treatment [7].

Diabetes promotes inflammation in the nerve tissues [8] that manifests symptoms and augments neuropathy development. Diabetic nerves contain macrophages, rarely lymphocytes and release increased tumor necrosis factor α (TNF‐α) or interleukins in humans and animals [9]. The proinflammatory state activates the stress kinase mitogen activated protein (MAP) kinase in diabetic nerves, which was reduced by pioglitazone treatment [10,11]. As a result, MAP kinase is evaluated as a potential target for a new diabetic neuropathy treatment. Nuclear factor NF-κB pathway was also triggered in this process, causing the cell to die or proliferate [12,13].

The previous works in the microvascular complications elucidates the clinical studies and drug-based progression of the diseases. The clinical studies to evaluate the presence of nephropathy and neuropathy in patients with DR and correlate the severity of DR with that of DN and DPN were studied [14]. Epalrestat, an aldose reductase inhibitor that is approved in Japan, prevented progression of diabetic neuropathy and retinopathy/nephropathy. The effect on diabetic retinopathy/nephropathy may have occurred indirectly because of the prevention of progression of diabetic neuropathy, in addition to the inhibitory action of epalrestat on aldose reductase [15].

The goal of this experiment was to perform WGCNA (Weighted Gene Correlation Networks Analysis) on DR, DN, and DPN samples in order to comprehend the disease's pathogenic pathways, and to find prospective biomarkers targets. As a result, RNA-sequencing (RNA-Seq) datasets from all three conditions were obtained from the European Nucleotide Archive (ENA)-European Bioinformatics Institute (EBI) database and significant differentially expressed genes (DEGs) were revealed. The samples were then introduced to WGCNA in order to construct the co-expression modules. The genes that overlapped in both the co-expressed modules and the DEGs were found. The biological linkages of overlapping genes in the development of all three microvascular problems were revealed by gene ontology (GO) and pathway enrichment analysis. The primary pathways involved in the pathophysiology of the complications of the gene of interest were discovered. The co-expressed genes that are differentially expressed in all three circumstances were identified for further investigation. The genes of interest’s protein-protein interaction (PPI) networks are constructed. A multi-layer regulatory network comprised of hub gene interrelationships, anticipated miRNAs, and transcription factors (TFs) and drugs targeting the genes were created and examined in order to uncover other regulatory agents impacting the expression of key genes.

**Fig. 1.**
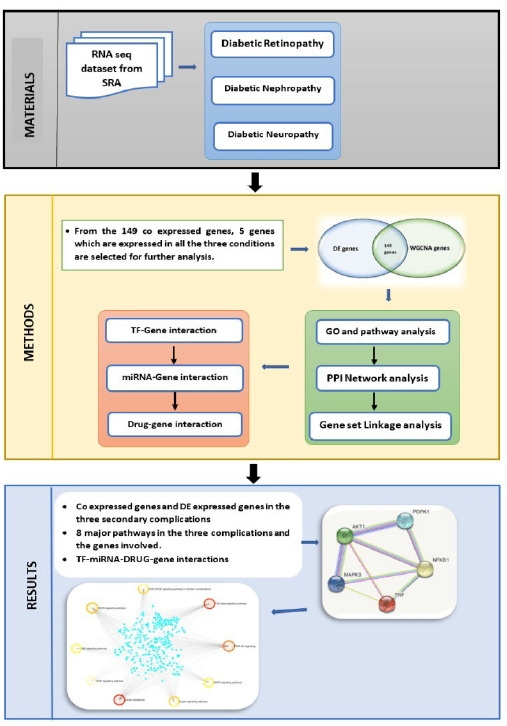
Workflow. SRA, Sequence Read Archive; TF, transcription factor; GO, gene ontology; PPI, protein-protein interaction; DE, differential expression.

This work focuses on the co-expression analysis of the three primary microvascular complications (nephropathy, retinopathy, and neuropathy), and to the best of our knowledge this is the first work focusing on all the three primary microvascular complications. In this study genes co-expressed in all the three disorders were first identified. The results were further enhanced to identify the TF, and miRNA that are commonly associated. The workflow is elucidated in Fig. 1. The final findings aid in pinpointing a single area that may be controlled or treated for all three disorders.

## Methods

### Data sources

The RNA-Seq expression profiles of three secondary complications from human were chosen for the study. Diabetic nephropathy - PRJNA595590 (36 samples – 28 DN and 9 control), diabetic neuropathy - PRJNA602424 (15 samples – 8 diabetic peripheral neuropathy samples and 7 normal controls), and diabetic retinopathy - PRJNA363068 (13 samples – 9 DR and 4 normal) were downloaded from ENA database (https://www.ebi.ac.uk/ena/browser/home). DN and DR are paired end samples whereas DPN is single end samples.

### Differential expression analysis

The RNA-Seq datasets of the three diabetes secondary complications were then examined for DEGs using DeSeq2 [16]. DEGs are genes that meet the requirements of |log fold change (FC)| > 1.5 and adjusted p-value < 0.05. The Venn diagram web tool (http://bioinfogp.cnb.csic.es/tools/venny/) was used to identify genes that were commonly seen in all three conditions. Using the approaches described above, a list of possible DEGs was compiled.

### Weighted Gene Co-expression Analysis

WGCNA in R [17] was used to identify co-expressed genes in DR, DN, and DPN. The normalized count files of all the samples were used as the input matrix. The co-expression network was built using the WGCNA algorithm. WGCNA is employed in sample clustering, topological feature calculation, co-expression network creation, disease-linked gene and module selection, and network differential analysis. Prior to WGCNA, outlier samples were discovered and removed using the principal component analysis method. The WGCNA algorithm was then given a matrix comprising the intensities of DEGs for each sample. Sample clustering, mean connectivity and scale-free fit index for numbers 1–30 (as soft-threshold power) were calculated individually with the best result determining the co-expression similarity of the adjacency matrix. Finally, using hierarchical clustering and TOM dissimilarity measures, all genes were classified into distinct modules (co-expression modules) based on their expression similarity. In this stage, after identifying module eigengenes (using pearsons correlation, r), the ones with significantly associated eigengenes (r > 0.85) were consolidated into a single module. According to the author, the following parameters were utilized to identify co-expression modules: "soft-threshold power = 12 min." CutHeight equals 0.15 when Merge ModuleSize = 30.

### Gene ontology and pathway enrichment of DEGs analysis

The ToppGene (ToppFun) (https://toppgene.cchmc.org/enrichment.jsp) [18] programme incorporated GO analysis (http://geneontology.org/) with Reactome (https://reactome.org/) [19] pathway enrichment analysis. As a result, GO analysis in ToppGene provides detailed annotations for functional and route interpretations of the gene of interest. DEGs were uploaded to ToppGene for GO and Reactome pathway enrichment analysis. p < 0.05 was chosen as the cut off criterion. The function of DEGs at three levels: molecular function, biological process, and cellular component were predicted from StringDB [20].

### Topology and PPI network analysis

Jetpetto in Cytoscape was used for topological analysis. StringDB interactome (https://string-db.org/) is a database of PPIs [20]. All candidate DEGs were uploaded in STRING, with a confidence score of 0.4 chosen as the cut off parameter for PPI network building. Then, using Cytoscape (version 3.9.0, http://www.cytoscape.org/) [21] software, a protein interaction association network was constructed and scaled in terms of node degree, betweenness centrality, stress centrality, and closeness centrality. Three modules of the highest degree were selected, and the probable mechanisms of each module were explored using ToppGene. The filter criterion was set to a degree of 10. Hub genes with a high degree of similarity were chosen as prospective important genes and biomarkers.

### Gene set linkage analysis of the genes

The gene set linkage analysis (GSLA) tool in Human Interactome Resource (HIR) was used to analyze the gene sets of interest [22]. The higher the density greater the link of the gene to the pathway or network. HIR is created by combining six sources of evidence for functional gene connections from nine public datasets. The GSLA tool is offered to analyze the probable functional impacts of the numerous simultaneously altered genes based on this high-quality functional association network of HIR. The gene set of interest is compared to a gene association network that includes the same genes and has the same number of neighbors and filtered with density more than 0.01.

### TFs-gene interaction in the specific pathways

The NetworkAnalyst database (https://www.networkanalyst.ca/) [23] is a free and open-source platform that focuses on TF–target interactions. ENCODE (Encyclopedia of DNA Elements), JASPAR, and ChEA are three well-known TF–target prediction databases used by NetworkAnalyst. Based on the ENCODE database, TFs were considered for the selected genes in this study. Cytoscape software was then used to visualize the network of hub genes and their targeted TFs.

### MiRNA-gene interaction

The miRNet database (https://www.mirnet.ca/) [24] is a free and open-source platform that primarily focuses on miRNA-target interactions. TarBase, miRTarBase, miRecords, miRanda, miR2Disease, HMDD, PhenomiR, SM2miR, PharmacomiR, EpimiR, starBase, TransmiR, ADmiRE, and TAM 2.0 are the 14 established miRNA-scape software was then used to show the network of the gene of interest and their targeted miRNAs.

### Drug-gene interaction

The Drug Bank database (version 5.0), PubChem, and Clue.io are used to investigate drug-gene interactions of the five genes of interest. Drugs were selected using the drug-gene interaction database (DGIdb) based on a selected gene of interest that acts as an exciting and prospective target [25]. This final drug list included only pharmaceuticals that have been approved by the Food and Drug Administration and had a DrugBank source.

## Results

### Differential expression analysis

**Fig. 2.**
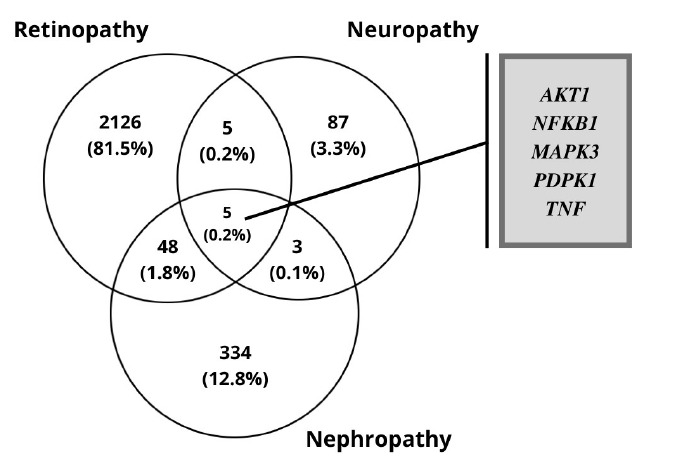
The overlapping differentially expressed genes of the three microvascular complications.

The RNA-Seq datasets of DR, DN, and DPN was obtained from the ENA-EBI. The identification of DEGs was processed with adjusted p < 0.05 and log FC > 1.5. A total of 2,184 genes were finally obtained including 1,949 up-regulated and 235 down-regulated genes in the DR dataset. In diabetic neuropathy 95 genes were obtained which includes 93 up-regulated and two down-regulated genes as listed in Table 1. The resulted DEGs of each dataset were subjected to Venn diagrams for the identification of overlapped genes among three conditions (Fig. 2). A total of 56 overlapped genes were identified. Forty-eight genes were found to be common in both DN and DR, five genes were found common for both neuropathy and retinopathy, and three genes were found to be overlapping in DN and DPN (Table 2, Fig. 2).

### Weighted gene co-expression analysis

**Fig. 3.**
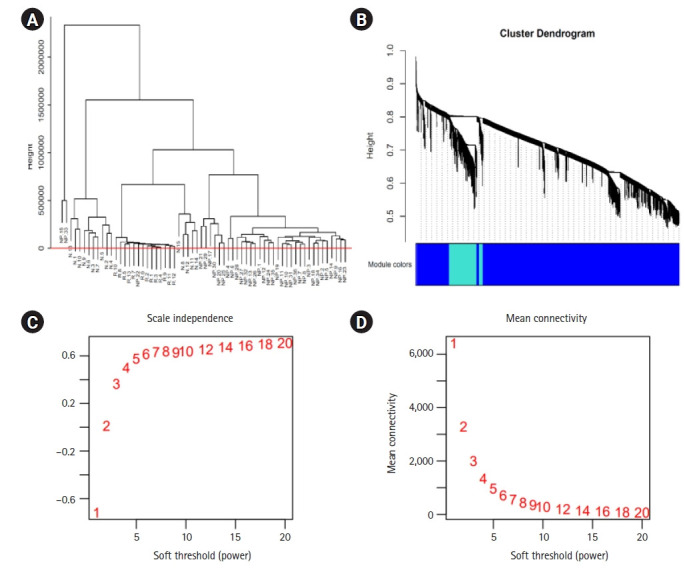
(A, B) The dendogram tree of the samples in the study clustering dendrogram of genes, with dissimilarity based on topological overlap. N, neuropathy; R, retinopathy; NP, nephropathy samples. (C, D) Analysis of network topology for various soft-thresholding powers. The left panel presents the scale-free fit index (y-axis) as a function of the soft-thresholding power (x-axis). The right panel displays the mean connectivity (degree, y-axis) as a function of the soft-thresholding power (x-axis).

**Fig. 4.**
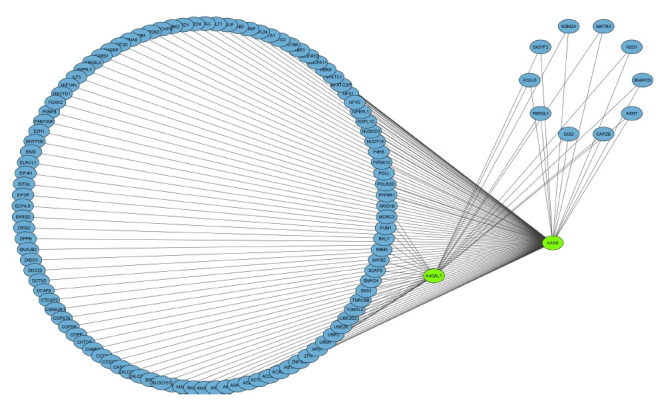
Co-expressed gene network of the 149 genes based on the weight.

The co-expressed genes are identified using WGCNA in R. Sample clustering showed no outlier among samples and soft-threshold power of 12 was selected based on the scale-free fit index and mean connectivity values. WGCNA algorithm clustered genes into six co-expression modules, including black, blue, turquoise, grey, dark-green, and light cyan modules. According to the eigengene’s clustering dendrogram and adjacency heatmap, six co-expression modules were divided into two clusters. Maximum number of genes falls into the blue and turquoise modules (Fig. 3B). Total interactions found was 65,535, after filtering the interactions with a cutoff of >0.4. The genes are filtered by mapping on to the DEGs—2,608, thereby getting 149 genes for further analysis. These 149 genes are further spotted for their interactions and network was generated. *A4GALT* and *AAAS* are the two major genes that show maximum interactions (Fig. 4). *AAAS* was found to be up-regulated in DN and has the highest degree of interactions in WGCNA analysis. *AAAS* was found to interact with *MAPK3* and *PDPK1*. *A4GALT* was found to be interacting with *PDPK1* and *AKT1*.

**Fig. 5.**
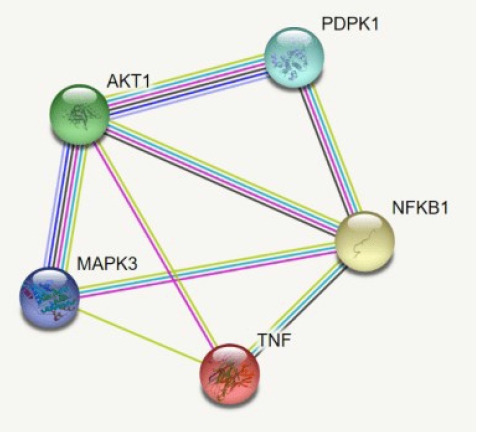
The interaction of the *AKT1*, *NFKB1*, *MAPK3*, *PDPK1*, and *TNF*.

The 149 DEG and co-expressed in WGCNA analysis were studied for the up or down regulation in three conditions. *AKT1*, *NFKB1*, *MAPK3*, *PDPK1*, and *TNF* were the five genes showing expression in all three conditions whereas all the other genes showed expression in either one or two conditions, thereby its excluded (Table 3,Fig. 5).

### GO and pathway enrichment analysis of 149 genes

In order to clarify the major functions of these DEGs, the associated biological processes were explored. GO enrichment and Reactome pathways analysis of DEGs were performed to analyze the gene function (in terms of biological processes, cellular components, and molecular function) as well as their associated pathways. GO enrichment analysis of top ten significantly enriched terms showed that in biological process category, the genes involved are concerned cellular process, metabolic process, organic substance metabolic process, nitrogen compound metabolic process, macromolecule metabolic process, and regulation of macromolecule metabolic process. In terms of cellular component, the genes were enriched in intracellular organelle, intracellular membrane-bounded organelle, cytoplasm, nucleus, intracellular organelle lumen, and nuclear lumen. For molecular function, category the genes were mainly concentrated in the heterocyclic compound binding, organic cyclic compound binding, nucleic acid binding, RNA binding and transcription co-regulator activity.

**Fig. 6.**
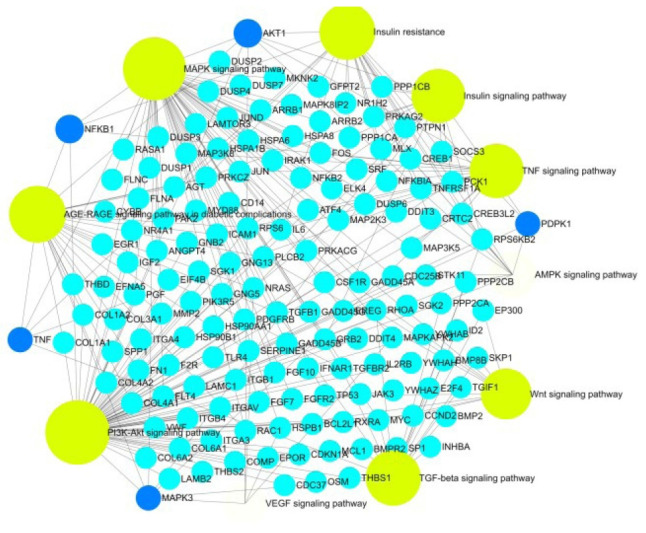
The major pathway (yellow) and the genes (blue).

From a total of 435 pathways, 121 pathways (p < 0.05) were obtained. Among the enriched pathways, eight pathways significant in diabetes were selected for further analysis. The major pathways and the respective gene of interest are given in Table 4. Nine major pathways in diabetes and its microvascular complications are considered for the study where all the five gene of interest is spotted. Reactome enrichment pathway analysis revealed that genes were significantly enriched in insulin resistance, phosphoinositide 3-kinase (PI3K)-Akt signaling pathway, TGF-β signaling pathway, mitogen-activated protein kinase (MAPK) signaling pathway, insulin signaling pathway, TNF signaling pathway, vascular endothelial growth factor (VEGF) signaling pathway, AGE-RAGE (receptor for advanced glycation end products) signaling pathway in diabetic complications and AMP-activated protein kinase (AMPK) signaling pathway (Fig. 6).

### Topology and PPI network analysis

**Fig. 7.**
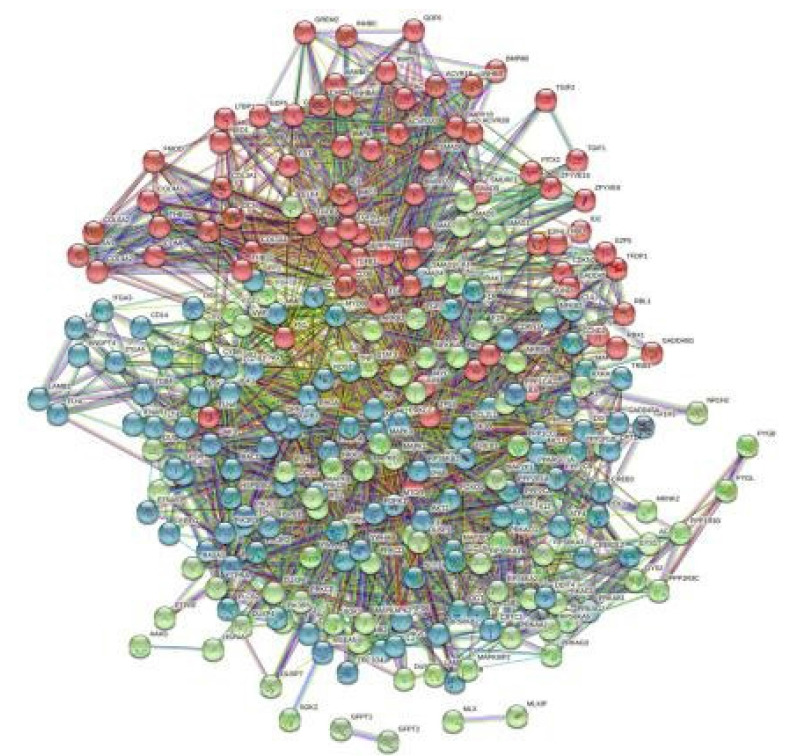
The three clusters of the 149 genes.

**Fig. 8.**
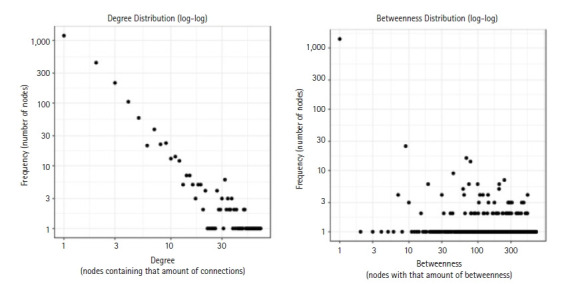
The degree distribution and betweeness distribution of the 149 genes.

By using the STRING database, the PPI network of 149 DEGs and co-expressed genes were established and consisted of 2,660 nodes and 5,066 edges (Fig. 7). Three clusters were obtained by k means clustering of the 149 genes. The study concentrates on *AKT1*, *NFKB1*, *MAPK3*, *PDPK1*, and *TNF* genes. The five genes of interest were found in two clusters. From the 111 genes interacting in the first cluster 73 genes from our 149 gene of interest are found to be interacting, *AKT1*, *TNF*, *PDPK1*, and *NFKB1* from the hub genes are found to be interacting in cluster 1 (blue). In the green cluster (second cluster) 43 of the key genes out of the 80 genes were found to be clustered. MAPK3 was found in the green cluster. In the third cluster (red), among the 74 genes interacting in the cluster, 31 key genes were found to be interacting. The degree distribution and betweeness distribution of the 149 genes on a logarithmic scale, shows a small number of nodes with high degree (the hubs) and a large number of nodes with a low degree (Fig. 8).

**Fig. 9.**
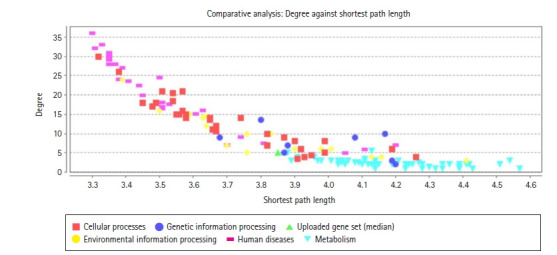
The topological segregation of the gene.

Kyoto Encyclopedia of Genes and Genomes (KEGG) pathway analysis of the selected modules revealed that the five gene of interest were identified in the major pathways such as insulin resistance, PI3K-Akt signaling pathway, TGF-beta signaling pathway, MAPK signaling pathway, insulin signaling pathway, TNF signaling pathway, VEGF signaling pathway, AGE-RAGE signaling pathway in diabetic complications and AMPK signaling pathway. Topology analysis elucidates from the selected 149 genes, most of the genes are spotted in metabolism. Diabetes being a metabolic disorder (Fig. 9).

### GSLA of the genes

The GSLA of the 149 genes was performed using GSLA tool in HIR. HIR is prepared through the integration of functional gene associations from nine public databases. Based on this high-quality functional association network of HIR, the GSLA tool interprets the potential functional impacts of the multiple simultaneously changed genes. The gene set of interest is compared to the gene association network consisting the same genes and having the same number of neighbors. TNF signaling, insulin receptor signaling cascade, VEGF signaling pathway, PIP3/AKT signaling, and MAPK1/MAPK3 signaling are the major pathways with high density and highest interaction. The genes of interest in these pathways are elucidated in Table 5. The density is higher the more the association of the gene to the pathway or network. Density considered was >0.01.

### TFs-gene interaction in the specific pathways

**Fig. 10.**
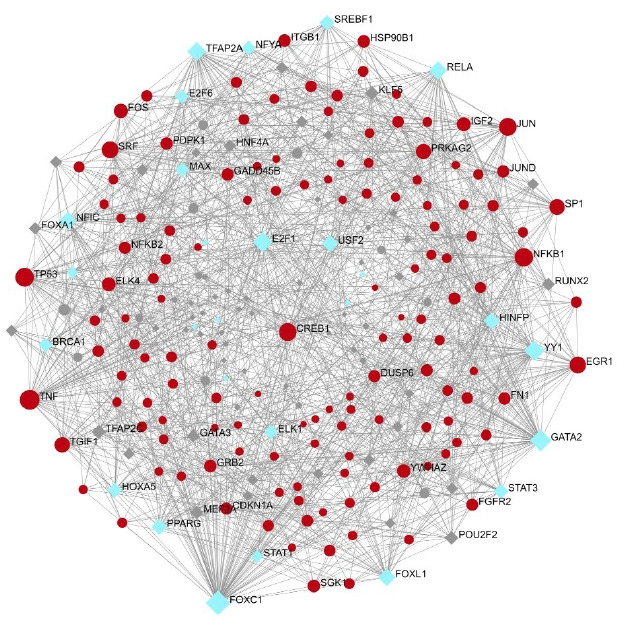
Transcription factor (blue) interacting with the gene of interest (red).

A total of 557 nodes and 8,063 edges of the 149 key genes were examined using Network analyst software. Subsequently, the resulted network was imported to Cytoscape for visualization of interaction among TFs and hub genes (Fig. 10). The top ranked TFs were RELA, PPARG, SREBF1, BRCA1, MAX, STAT1, HNF4A, PDX1, MYB, NFATC2, and FOXO3 are shown in Table 6. Based on the results, we found that degree level of RELA was very high. It was co-regulated by all the five gene of interest and many other TFs, which fall into the major pathways. Subsequently, the network of the hub genes and their targeted TFs were visualized by Cytoscape software. RELA, FOXO3, PDX1, and SREBF1 were the TFs within highest degree and interacting with the major gene of interest (Table 7).

### miRNA-gene interaction

**Fig. 11.**
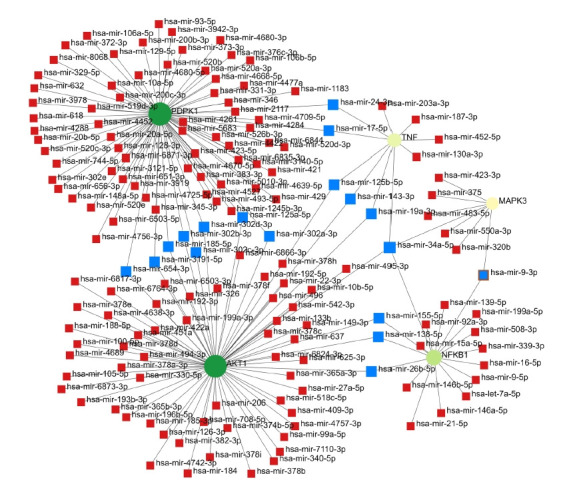
Red nodes are miRNAs and blue nodes are the highly interaction miRNAs.

A total of 2,463 nodes and 10,246 edges of the 149 key genes and their miRNA interactions were examined from miRNet database. The list of miRNA interacting with the five gene of interest was further examined (Table 8). miRNA-hub gene regulatory network construction miRNet database was applied to screen the targeted miRNAs of the hub genes. Cytoscape software was used to construct the miRNA-hub gene network (Fig. 11). Subsequently, the network of the five genes and their targeted miRNAs was visualized by Cytoscape software. hsa-mir-34a-5p is the major miRNA which is found to interact with all the five genes (Table 8).

### Drug-gene interaction

**Fig. 12.**
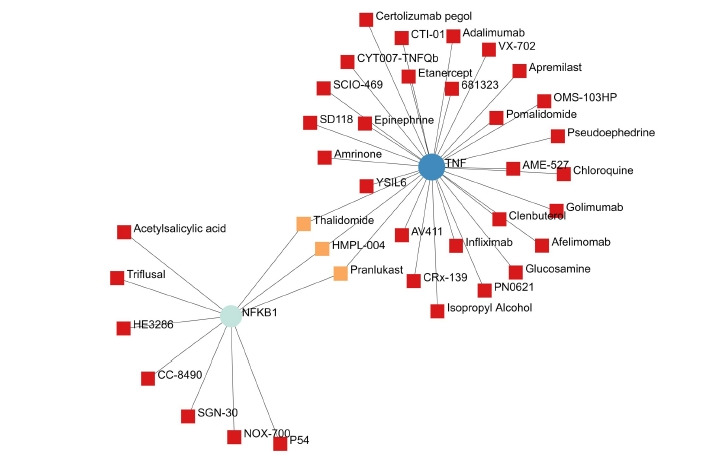
The *NFKB1* and *TNF* gene and their drugs.

**Fig. 13.**
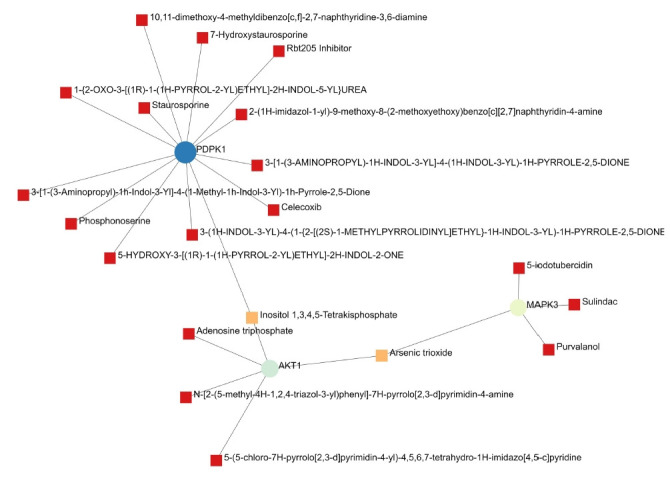
The *PDPK1*, *AKT1*, and *MAPK3* gene and their drugs.

A total of 69 drugs were explored using DGIdb that might have the potential to treat the major five genes. Furthermore, downstream interaction networks of *AKT1*, *NFKB1*, *MAPK3*, *PDPK1*, and *TNF* were generated (Figs. 12, 13), which elucidates the common drugs interacting with one or more than one gene of interest. Thalidomide, HMPL-004 (Andrographolide) and Pranlukast were found to be targeting *TNF* and *NFKB1*. Inositol 1,3,4,5-tetrakisphosphate was found to target both *PDPK1* and *AKT1*. Arsenic trioxide was targeting *AKT1* and *MAPK3* form the five gene of interest was studied in DrugBank source (Table 9).

## Discussion

DM produces vascular disease that accounts for the majority of morbidity, hospitalizations, and deaths among patients. Microvascular lesions induce nephropathy, retinopathy, and neuropathy, whereas major blood vessel damage raises the risk of cardiovascular events. The onset of a secondary diabetic complication cannot be avoided or reversed. The study focused on co-expressed genes in all three conditions (DR, DN, and DPN), pathogenesis, and miRNA, TF and gene network in the key pathways involved. In the DR dataset, the differential expression analysis of all three complications includes a total of 2,184 genes, in which 1,949 up-regulated and 235 down-regulated genes. DN qualifies 330 genes to be up-regulated and 55 genes to be down-regulated. Diabetic neuropathy was found to have 95 genes, 93 of which were up-regulated and two were down-regulated. A total of 56 overlapping genes were identified, 48 genes were found to be common in diabetic neuropathy and retinopathy, five genes were found to be common in neuropathy and retinopathy, and three genes were found to be overlapping in diabetic neuropathy and nephropathy.

After filtering the interactions with a cut off of >0.4, a total of 65,535 interactions were obtained from the co-expression analysis using WGCNA. By mapping the genes to the DEGs – 2,608, a total of 149 genes were obtained for further study. The relationships between these 149 genes were identified, and a network was created. The two primary genes with the most interactions are *A4GALT* and *AAAS* (Fig. 4). *AAAS* has the highest degree of interactions in WGCNA analysis and has been discovered to be up-regulated in DN. *AAAS* has been discovered to interact with *MAPK3* and *PDPK1*. *A4GALT* has been discovered to interact with *PDPK1* and *AKT1*. In order to identify the genes most often related with disease and their roles, it is helpful to identify the genes with co-expressed profiles.

The up or down regulation of the 149 DEG and co-expressed in WGCNA analysis were investigated in three disease condition. The five genes *AKT1*, *NFKB1*, *MAPK3*, *PDPK1*, and *TNF* that exhibited expression in all three conditions were considered for further analysis. All the other genes that showed expression only in two or one condition. Diabetes-prone mice's blood glucose levels were reported to be controlled by MAPK3 (ERK1) inhibitor inhibition in hypertrophic 3T3-L1 [26,27]. In a study involving DN and non-DN patients, it was found that addition to poor glycemic control, oxidative stress and inflammation; genetic factors seem to be main determinants of DN in terms of both occurrence and severity [28]; however, the genetic mechanism causing DN is still unexplored. When compared to people with type 2 diabetes mellitus without nephropathy, subjects with DN had higher levels of uMCP-1 and plasma TNF, and they found a significant link between uMCP-1 and plasma TNF. Gupta et al.’s study [29] has also emphasized the connection between DN and the single nucleotide polymorphism of the TNFA gene.

The study takes into account nine key pathways in diabetes and associated microvascular complications, with all five genes of interest being identified. Insulin resistance, PI3K-Akt signaling pathway, TGF-beta signaling pathway, MAPK signaling pathway, insulin signaling pathway, TNF signaling pathway, VEGF signaling pathway, AGE-RAGE signaling pathway in diabetic complications, and AMPK signaling pathway were all found to be significantly enriched in the reactome enrichment pathway analysis.

Two major categories of regulatory elements that control gene expression are TFs and miRNAs. Table 6 shows the top TFs: RELA, PPARG, SREBF1, BRCA1, MAX, STAT1, HNF4A, PDX1, MYB, NFATC2, and FOXO3. Based on the findings, we discovered that the interaction degree of RELA was extremely high, as it was co-regulated by all five genes of interest as well as a slew of other TFs involved in significant pathways. Following that, Cytoscape software was used to visualize the network of hub genes and their targeted TFs. RELA, FOXO3, PDX1, and SREBF1 are the TFs that possess highest interaction with the primary gene of interest. NF-κB is a widely distributed TF that has a role in a variety of biological processes. Specific inhibitors keep it in an inactive condition in the cytoplasm. NF-κB travels to the nucleus after the inhibitor is degraded and promotes transcription of certain genes. *NFKB1* or *NFKB2* are linked to REL, RELA, or RELB to form NF-κB. The most common form of NF-κB is *NFKB1* complexed with RELA, the gene's product. This gene has four transcript variants that code for distinct isoforms. In our study, RELA interacts with *NFKB1*, which is the most common form of complex. This complex is not studied in the three diabetes secondary complications [30]. Insulin inhibits FoxO TFs, making them important insulin action mediators in diabetic mice. It has been established from the literature that FoxO-regulated genes are rate-limiting in the enhanced protein breakdown and muscle atrophy seen in diabetes with insufficient insulin [31].

The construction of a miRNA-hub gene regulation network revealed that 22 significant miRNA interact with the five genes of interest. The primary miRNA observed to interact with all five genes is hsa-mir-34a-5p. miR-34a-5p could negatively influence pancreatic cell proliferation via the Wnt signaling pathway using GO and KEGG enrichment analysis. It was also discovered to affect blood glucose levels via regulating insulin secretion via the insulin signaling system in their study [32]. Gholaminejad, Gholaminejad et al. (2021) [33], in their findings states that hsa-miR-129-2-3p, hsa-miR-34a-5p, and hsa-miR-27a-3p, as well as STAT3, were identified as top molecules directing the regulation of the hub genes in the created regulatory network in immunoglobulin A nephropathy. MicroRNA-34a-5p (miR-34a) has been involved in vascular senescence [34], oxidative stress [35] and apoptosis [36] as a translational inhibitor of SIRT1. MiR-34a expression is altered in a variety of human diseases, including cancer [38]; and cardiovascular disease [38]. From previous literatures, hsa-miR-34a-5p was found to be a prominent biomarker in diabetes and not found to be proved in DR, DN, and DPN. In our present study, hsa-miR-34a-5p was found to be interacting with five gene of interest in the major pathways in the selected diabetes secondary complications. miR-34a-5p and RELA were identified through the construction of a regulatory network as putative top molecules found to be possibly regulating the expression of the five identified gene of interest.

Additionally, downstream drug-gene interaction networks for *AKT1*, *NFKB1*, *MAPK3*, *PDPK1*, and *TNF* were created (Figs. 12, 13), revealing the common medications that interact with one or more genes of interest. *TNF* and *NFKB1* were discovered to be targeted by Thalidomide, HMPL-004 (Andrographolide), and Pranlukast (Fig. 12). Both *PDPK1* and *AKT1* have been discovered to be targets of inositol 1,3,4,5-tetrakisphosphate (Fig. 13). The five gene of interest, *AKT1* and *MAPK3*, were targeted by arsenic trioxide.

This study elucidates the key co-expressed genes, which are up or down-regulated in all the three microvascular complications. The most prominent interaction of miRNA, TF, and gene in the major pathways of the diabetes and its complications elucidates a better understanding to the pathogenesis of the diseases. Thereby proposing the most potential biomarkers in the regulation or prevention of the diabetes secondary complications that affect the kidney, eye and nerves.

Despite the fact that several of the aforementioned strategies in this study, have produced positive outcomes for discovering new information, there are still certain restrictions. In our case, data on DN, neuropathy, and retinopathy were collected using Illumina HiSeq 4,000, 3,000, and 2,000. Data loss may have occurred as a result of the experimental platforms' differences. The differences and lack of genes won't hinder the research, though, because each of the three disorders was studied separately. For researchers who seek to comprehend the related pathways involved in the pathophysiology and progression of all the three microvascular complications, our research will be a crucial pioneer. Our results thus have the potential to improve future treatment of diabetes complications by identifying particular biological pathways and genes linked to each type of complication.

This was the first study to construct a co-expression network to explore the three diabetes-associated secondary complications—DR, DN, and DPN. Our findings revealed five key genes that acted as essential components in the etiology of diabetes-associated microvascular complications, which may enhance our fundamental knowledge of the molecular mechanisms underlying this disease. The TF RELA and miRNA-hsa-mir-34a-5p interacts with all the five genes *AKT1*, *NFKB1*, *MAPK3*, *PDPK1*, and *TNF*. They play a major role in progression of pathogenesis of the three diseases. Hence, these genes might act as potential biomarkers for diagnosis of both diseases at early stage. Our findings will pave the way for further research into the related pathways that play a role in the development and pathophysiology of both diseases.

## Figures and Tables

**Table 1.** The up- and down-regulated gene of the three microvascular complications

	Diabetic retinopathy	Diabetic neuropathy	Diabetic nephropathy
Up-regulated	1,949	93	330
Down-regulated	235	2	55

**Table 2.** The differentially expressed genes overlapping in the three conditions

Nephropathy and retinopathy				Nephropathy and neuropathy	Neuropathy and retinopathy
*MAP1LC3B*	*NIPSNAP3B*	*PTPRE*	*INSC *	*TPRG1*	*ARL4C*
*PTPN1*	*FCGR2A*	*MXRA5*	*EPPIN-WFDC6*	*CERKL*	*PMAIP1*
*SNAPIN*	*CEP250*	*GNS*	*GPNMB*	*PCK1*	*SLC7A11*
*MAP6D1*	*TPRG1L*	*RNASE1*	*PDCL*		*NDST3*
*PRPH2*	*TMC8*	*TKT*	*ST8SIA4*		*PTGFR*
*SCG3*	*DEGS1*	*RAB11A*	*PER1*		
*UBA7*	*STARD4*	*THBD*	*NR4A1*		
*RPS6KB2*	*MARCKSL1*	*TYROBP*	*FOS*		
*PSMB4*	*POLG2*	*SLC43A3*	*EGR1*		
*RPS11*	*COX6C*	*HMGN4*	*RASGEF1B*		
*RGS19*	*SRSF10*	*C15orf48*	*HSPA8*		
*TUBB3*	*ITGB2*	*ELOVL3*	*PPP2CB*		

**Table 3.** The expression of the selected genes in three conditions

S. No.	Gene	Retinopathy		Neuropathy		Nephropathy	
		Up	Down	Up	Down	Up	Down
1	*AKT1*	Y	N	Y	N	Y	N
2	*NFKB1*	Y	N	N	Y	Y	N
3	*MAPK3*	Y	N	Y	N	N	Y
4	*PDPK1*	Y	N	Y	N	Y	N
5	*TNF*	Y	N	Y	N	N	Y

**Table 4.** The major enriched pathway and their gene of interest

S. No.	Pathway	Genes of interest	p-value
1	PI3K-Akt signaling pathway	*AKT1*	1.75E-103
		*NFKB1*	
		*MAPK3*	
		*PDPK1*	
2	MAPK signaling pathway	*AKT1*	5.47E-83
		*NFKB1*	
		*MAPK3*	
		*TNF*	
3	AGE-RAGE signaling pathway in diabetic complications	*AKT1*	7.46E-35
		*NFKB1*	
		*MAPK3*	
		*TNF*	
4	TGF-beta signaling pathway	*MAPK3*	4.28E-28
		*TNF*	
5	TNF signaling pathway	*AKT1*	5.88E-27
		*NFKB1*	
		*MAPK3*	
		*TNF*	
6	AMPK signaling pathway	*AKT1*	5.97E-25
		*PDPK1*	
7	Insulin signaling pathway	*AKT1*	2.69E-28
		*NFKB1*	
		*PDPK1*	
		*TNF*	
8	Insulin resistance	*AKT1*	5.21E-25
		*MAPK3*	
		*PDPK1*	
9	VEGF signaling pathway	*MAPK3*	1.22E-13

PI3K, phosphoinositide 3-kinase; MAPK, mitogen-activated protein kinase; AGE, advanced glycation end-products; RAGE, receptor for advanced glycation end products; TGF, transforming growth factor; AMPK, AMP-activated protein kinase; VEGF, vascular endothelial growth factor.

**Table 5.** The five genes and the linkage density to the respective pathway

S. No.	Description	Density	Interaction number	Overlap gene number	Overlapped gene(s)
1	TNF signaling	0.03233	212	2	*TNF*
2	PIP3 activates AKT signaling	0.03029	1,160	22	*AKT1/PDPK1/MAPK3*
3	MAP2K and MAPK activation	0.06191	369	8	*MAPK3*
4	AKT phosphorylates targets in the cytosol	0.06833	112	2	*AKT1/CDKN1A*
5	Insulin receptor signaling cascade	0.04171	317	6	*MAPK3*
6	Insulin-like growth factor I binding	0.05857	96	2	*ITGAV/ITGB4*
7	VEGF signaling pathway	0.06469	588	7	*AKT1/MAPK3*
8	MAPK1/MAPK3 signaling	0.02869	1,227	27	*MAPK3*
9	IGF factor receptor signaling pathway	0.07046	105	1	*AKT1*
10	Negative regulation of insulin receptor signaling pathway	0.04785	164	1	*PRKCZ*
11	VEGFA-VEGFR2 Pathway	0.0563	797	14	*PDPK1/AKT1*
12	Insulin/IGF pathway-MAPK kinase/MAPK cascade	0.08817	381	6	*MAPK3*
13	Insulin/IGF pathway-protein kinase B signaling cascade	0.05119	267	4	*AKT1/PDPK1*
14	PI3K cascade	0.0305	200	5	*PDPK1*
15	Signaling by insulin receptor	0.02979	333	6	*PDPK1/MAPK3*
16	Signaling by VEGF	0.05317	824	16	*PDPK1/AKT1*

*TNF*, tumor necrosis factor; MAPK, mitogen-activated protein kinase; VEGF, vascular endothelial growth factor; MAP, mitogen-activated protein; IGF, Insulin-like growth factor; PI3K, phosphoinositide 3-kinase.

**Table 6.** The top ranked TFs and their degree and betweenness

S. No.	TF	Degree	Betweenness
1	RELA	38	837.07
2	PPARG	26	389.71
3	SREBF1	24	298.59
4	BRCA1	21	248.06
5	MAX	20	199.62
6	STAT1	15	152.47
7	HNF4A	14	107.66
8	PDX1	4	7.83
9	MYB	1	0
10	NFATC2	1	0
11	FOXO3	1	0

TF, transcription factor.

**Table 7.** Top ranked TFs: gene interaction in the major nine pathway

S. No.	Pathway	TF	Gene of interest
1	PI3K-Akt signaling pathway	MYB, RELA, FOXO3, BRCA1	*AKT1, NFKB1, MAPK3, PDPK1*
2	MAPK signaling pathway	RELA, MAX, ELKI	*AKT1, NFKB1, MAPK3, TNF*
3	AGE-RAGE signaling pathway in diabetic complications	RELA, STAT1	*AKT1, NFKB1, MAPK3, TNF*
4	Insulin resistance	RELA, PDX1, SREBF1, STAT3	*AKT1, NFKB1, TNF*
5	TGF-beta signaling pathway	PDX1	*MAPK3, TNF*
6	TNF signaling pathway	RELA	*AKT1, NFKB1, TNF*
7	AMPK signaling pathway	FOXO3, HNF4A, SREBF1, PPARG	*PDPK1, AKT1*
8	Insulin signaling pathway	SREBF1, PDX1, ELK1	*AKT1, MAPK3, PDPK1*
9	VEGF signaling pathway	PDX1, NFATC2	*AKT1, MAPK3*

TF, transcription factor; PI3K, phosphoinositide 3-kinase; MAPK, mitogen-activated protein kinase; AGE, advanced glycation end-products; RAGE, receptor for advanced glycation end products; TGF, transforming growth factor; *TNF*, tumor necrosis factor; AMPK, AMP-activated protein kinase; VEGF, vascular endothelial growth factor.

**Table 8.** The five gene of interest and miRNAs interaction

S. No.	Gene	miRNA
1.	*NFKB1, MAPK3, AKT1, TNF*	hsa-mir-34a-5p
2.	*NFKB1 & MAPK3*	hsa-mir-34a-5p, hsa-mir-9-3p
		3.	*MAPK3 & AKT1*	hsa-mir-17-5p, hsa-mir-24-3p
4.	*TNF & PDPK1*	
		hsa-mir-24-3p
5.	*TNF & AKT1*	hsa-mir-19a-3p, hsa-mir-125b-5p, hsa-mir-34a-5p, hsa-mir-143-3p
6.	*AKT1 & NFKB1*	hsa-mir-26b-5p, hsa-mir-138-5p, hsa-mir-34a-5p, hsa-mir-155-5p
7.	*PDPK1 &AKTI*	hsa-mir-125a-5p, hsa-mir-302c-3p
		hsa-mir-185-5p, hsa-mir-302d-3p
		hsa-mir-302a-3p, hsa-mir-654-3p
		hsa-mir-302b-3p, hsa-mir-3191-5p

**Table 9.** The gene of interest and their respective drugs

S. No.	Gene	Drug
1	*TNF & NFKB1*	Thalidomide
		HMPL-004 (andrographolide)
		Pranlukast
2	*PDPK1 & AKT1*	Inositol 1,3,4,5-tetrakisphosphate
3	*AKT1 & MAPK3*	Arsenic trioxide
